# Arsenic exposure and risk of nonalcoholic fatty liver disease (NAFLD) among U.S. adolescents and adults: an association modified by race/ethnicity, NHANES 2005–2014

**DOI:** 10.1186/s12940-017-0350-1

**Published:** 2018-01-15

**Authors:** Jennifer K. Frediani, Eric A. Naioti, Miriam B. Vos, Janet Figueroa, Carmen J. Marsit, Jean A. Welsh

**Affiliations:** 1 Department of Pediatrics, Emory University School of Medicine, Atlanta, GA USA; 2 Department of Biostatistics and Bioinformatics, Rollins School of Public Health, Emory University, Atlanta, GA USA; 3 Department of Environmental Health, Rollins School of Public Health, Emory University, Atlanta, GA USA; 4 Wellness Department, Children’s Healthcare of Atlanta, Atlanta, GA USA

**Keywords:** Nonalcoholic fatty liver disease (NAFLD), Arsenic, Obesity, NHANES, Hispanic, Alanine aminotransferase

## Abstract

**Background:**

While associated with obesity, the cause of the rapid rise in prevalence of nonalcoholic fatty liver disease (NAFLD) in children, which is highest among Hispanics, is not well understood. Animal experiments have demonstrated that arsenic exposure contributes to liver injury. Our objective was to examine the association between arsenic exposure and NAFLD in humans and to determine if race/ethnicity modifies the association.

**Methods:**

Urinary inorganic arsenic concentrations among those ≥12 years in the National Health and Nutrition Examination Survey, 2005–2014 were used to assess the cross-sectional association with serum alanine aminotransferase (ALT) levels, a marker of liver dysfunction. We excluded high alcohol consumers (>4–5 drinks/day; *n* = 939), positive hepatitis B or C (*n* = 2330), those missing body mass index (*n* = 100) and pregnant women (*n* = 629) for a final sample of 8518. Arsenic was measured using liquid chromatography coupled with mass spectrometry and ALT was measured using standard methods. Sampling weights were used to obtain national estimates. Due to lack of normality, estimates were log transformed and are presented as geometric means. Logistic regression models controlling for age, sex, income, and weight category estimate adjusted odd ratios (aOR) of elevated ALT by quartile of arsenic and tested for effect modification by race/ethnicity and weight. Elevated ALT was defined as >25 IU/L and >22 IU/L for boys and girls ≤17 years, respectively and >30 IU/L and >19 IU/L for men and women, respectively.

**Results:**

Among all, aOR of elevated ALT were higher among those in the highest vs. lowest arsenic quartile (referent), 1.4 (95% confidence interval [CI]: 1.1, 1.7) with a borderline significant interaction (*p* = 0.07) by race/ethnicity but not weight (*p* = 0.4). In analysis stratified by race/ethnicity, aOR of elevated ALT among those in the 4th quartile were higher among Mexican Americans, 2.0 (CI: 1.3, 3.1) and non-Hispanic whites only, aOR 1.4 (CI: 1.1, 1.8) despite the fact that obesity prevalence was highest among non-Hispanic blacks.

**Conclusions:**

Our findings demonstrate a positive association between urinary arsenic exposure and risk of NAFLD among U.S. adolescents and adults that is highest among Mexican Americans and among those obese, regardless of race/ethnicity.

## Background

The prevalence of nonalcoholic fatty liver disease (NAFLD), which is characterized by an accumulation of fat in the liver, has risen markedly in the U.S. over recent decades [1]. Currently prevalence is estimated to be 30% among adults in the U.S. [2] with rates highest among males and among Hispanic Americans, specifically Mexican Americans [1, 3]. Described as the liver manifestation of metabolic syndrome, NAFLD can progress to include liver inflammation and fibrosis and increase risk of liver cancer [4]. While NAFLD occurs primarily in the presence of obesity, its cause is unknown. The genetic variant, PNPLA3, which is common among Hispanics, has been associated with alanine aminotransferase (ALT) levels, a marker of liver damage, and increased hepatic fat [5–7]. Recent evidence suggests that exposure to environmental pollutants including those that are inhalation and ingestion toxicants [8–10], including heavy metals such as arsenic [11], may play a role. Lin et al. previously found a significant association between several soil-derived heavy metals, including arsenic, and NAFLD in lean Tiawanese men, but the independent effect of arsenic was not examined [11].

Arsenic is present in the environment in both organic and inorganic form. Exposure to the inorganic forms (iAs), arsenite and arsenate, is known to be carcinogenic, atherogenic, neuropathogenic. At low doses, iAs has also been shown to diminish insulin sensitivity in both children and adults and to induce diabetes in animals and humans [12–14]. Adults with trace increases in urinary iAs have 50% greater risk of type 2 diabetes [15]. When iAs concentrations in drinking water are low (< 10 μg/L), such as in the U.S., where they are regulated in public water supplies, foods such as rice, other cereals and cereal products, infant rice-based cereals and poultry are the major contributors. [16–18] iAs contamination in rice results from soil contamination. The rice plant extracts iAs from the soil and concentrates it in the grain. Previously iAs was also found in poultry due to the use of arsenic-based drugs to prevent histomoniasis in U.S. produced chicken and turkey. This practice was discontinued in 2016 [19]. Notably, urinary iAs concentrations in the U.S. are increased in Asians and Hispanics compared to non-Hispanic whites, presumably because of cultural dietary differences [20–22]. In U.S. children, for every 1/4 cup per day increase in rice consumption, urinary arsenic concentrations increased by 14% (CI 14–17%) [23].

In murine models, iAs invokes various changes to hepatic tissue at relatively low doses. It has been shown to change the vascularization of the liver tissue [24] and gene expression within hepatic transporters [25]. Tan and colleagues fed male C57BI/6 J mice either a high fat or low fat diet and exposed them to arsenic through drinking water. While the low fat diet mice did not have increased liver damage, the combination of high fat diet and iAs produced the highest degree of liver damage and inflammation [26]. These changes in conjunction with other risk factors such as obesity suggests that low dose iAs could play an important role in NAFLD.

Due to the known dysmetabolic effect of iAs exposure, we aimed to use data from a national sample to assess its association with NAFLD risk and to determine if this exposure may help to explain the race/ethnic differences in risk that have been observed. We hypothesized that greater exposure to arsenic may be contributing to the higher prevalence of NAFLD among Hispanics.

## Methods

### Study population

Data from the National Health and Nutrition Examination Survey (NHANES) was used in this study. NHANES is a cross-sectional survey with a complex, multi-stage, probability sampling design. Ten years of data across five NHANES cycles (2005/2006 through 2013/2014) were examined. In NHANES, arsenic exposure, through urinary metabolite analysis, was assessed in participants aged 12 years and older. We excluded from the sample participants with hepatitis B or C (*n* = 2330) and those with high alcohol consumption (>4–5 drinks/day; *n* = 939) given that these conditions can cause liver damage and raise ALT levels. We excluded those missing body mass index (BMI) data (*n* = 100) and those pregnant (*n* = 629) leaving a final sample of 8516. We created a separate category (*n* = 647) for those with missing poverty income ratio (PIR) but maintained them in the sample.

### Arsenic exposure

Urine samples were collected in arsenic free containers and were analyzed for total urinary arsenic concentrations. These measurements reflect approximately the last 3 days of exposure [27, 28]. Inductively coupled-plasma dynamic reaction cell-mass spectrometry (ICP-DRC-MS) was used to measure total arsenic and speciated arsenic, including arsenobetaine and arsenocholine, which are arsenosugars and are considered non-toxic [29]. The lower limit of detection (LLOD) for arsenobetaine was 0.4 μg/L for 2005 through 2010, 1.19 μg/L in 2011/2012, and 1.16 μg/L in 2013/2014. For arsenocholine, the LLOD was 0.6 μg/L for 2005 through 2010, 0.28 μg/L in 2011/2012, 0.11 μg/L in 2013/2014. For total arsenic and speciated arsenics, samples that were below the LLOD (*n* = 90 samples) were assigned a value of LLOD divided by the square root of two [30]. From total arsenic measures, total inorganic arsenic concentrations were estimated by subtracting measurements for arsenobetaine and arsenocholine. Due to measurement error, some estimates for arsenic (*n* = 105) were below zero (μg/L); therefore, any value below 0.01 (μg/L) was replaced by 0.01 [31]. Arsenic concentrations were corrected for urine dilution using a covariate-adjusted standardization of arsenic concentration divided by creatinine concentration to control for measurement error bias caused by urinary diluteness [31]. Urine samples were analyzed on a Beckman CX3 using the Jaffe reaction to quantify creatinine concentrations in the 2005/2006 cycle. From 2007 through 2014 creatinine was measured on a Roche ModP Chemistry Analyzer using an enzymatic method. Measurements for creatinine corrected iAs were stratified into quartiles based on estimates of population quartiles of 2.42, 4.08, and 6.99 (μg/L) for the first quartile, median, and third quartile respectively.

### NAFLD assessment

ALT, a liver enzyme often elevated in the presence of liver disease is commonly used as a screening test and monitoring biomarker for NAFLD [32–34]. Individuals age 12 years and older participating in NHANES had fasting blood samples taken at a mobile examination center. Serum ALT concentrations (U/L) were measured either on a Beckman Synchron LX20 (2005/2006) using an enzymatic rate method, or on a Beckman UniCel DxC800 Synchron (2007 through 2014) using a kinetic rate method. ALT was dichotomized use cut-offs commonly used clinically: high ALT was defined as >25 IU/L for boys ≤17 years and >22 IU/L for girls ≤17 years [32]. In adults, high ALT was defined as >30 IU/L in males and >19 IU/L in females [35].

### Other covariates

Demographic variables of age (in years), sex, race/ethnicity and household income, were collected via interviewer assisted interviews. The poverty-to-income ratio (PIR) was calculated as the annual household income divided by the poverty threshold determined annually by the U.S. Department of Health and Human Services and grouped into three categories: <130, 130–350, >350% for analysis [36]. Participants’ height and weight were measured at the NHANES mobile examination center. BMI was calculated as weight (kg)/height (m^2^) and used to group study participants as underweight (<18.5), normal weight (18.5- < 25), overweight (25- < 30) and obese (≥30).

Hepatitis B and C positive participants were defined as those testing positive for the hepatitis B core antibody (Anti-HBc) or hepatitis C antibody (Anti-HCV). The VITROS Anti-HBc and Anti-HCV assays were performed using the VITROS Anti-HBc or Anti-HCV Reagent Pack and the VITROS Immunodiagnostic Products Anti-HBc or Anti-HCV calibrator on the VITROS ECi/ECiQ or VITROS 3600 Immunodiagnostic System. Hepatitis C antibody data was not provided for the NHANES cycle from 2013 to 2014, therefore no participants were excluded from this cycle for having hepatitis C.

### Statistical analysis

Descriptive analyses and regression model fitting were conducted using methods that accounted for the complex survey design of NHANES. Since multiple cycles were used, weights were adjusted by dividing by the number of cycles in each weighted analysis. The distribution of iAs was right skewed and therefore log-transformed. ANOVA and post-hoc *t*-tests were used for comparisons between variables of interest. Geometric means were presented. (Tables 2 and 3) As the relationship between the log-transformed iAs and elevated ALT levels was not linear for some race/ethnic groups, iAs concentrations were analyzed in quartiles. Logistic regression was used to estimate adjusted odds ratios (aORs) of elevated ALT adjusting for age, ethnicity, sex, weight category, and PIR (Table 4) with increasing iAs concentrations (in quartiles) stratified by race/ethnicity and weight category. Survey year was also included as a categorical variable in the models to adjust for any temporal changes between survey cycles. This analysis was also repeated among those obese only and stratified by race/ethnicity. We assessed for interactions of weight status (categorized) and of race/ethnicity with quartiles of arsenic exposure by including multiplicative interaction terms in the full models.

SAS 9.4 (SAS Institute Inc., Cary, NC) was used for all analysis. A *p*-value of 0.05 was used to determine statistical significance. Sampling weights were applied to all analyses to obtain nationally representative estimates. A sensitivity analysis was done using unweighted data to confirm the stability of the weighted estimates.

## Results

The sample-weighted median age was 38 (interquartile range [IQR] 20, 58) years. Most were non-Hispanic white (42%) followed by non-Hispanic black (21%), Mexican-American (19%), other Hispanics (9%) and other/mixed (8%) (Table 1). Obesity prevalence was significantly higher among non-Hispanic blacks and significantly lower among the other/mixed race group compared to all other race/ethnic groups (not shown). Geometric mean ALT was 21.5 U/L (21.2, 21.8) with concentrations significantly higher among adults, males, Mexican Americans, other Hispanics, or non-Hispanic whites, higher income, and those overweight or obese. (Table 2) The geometric mean iAs was 3.8 mcg/L (3.5, 4.1) with concentrations higher among adults, males, other Hispanics and other/mixed race (Table 3). There were no significant differences in iAs by income level or weight category.

**Table 1:** Characteristics of NHANES 2005–2014 unweighted sample

Characteristics (*n* = 8518)	Value
Age (years) [median(Q1,Q3)]	38 (20, 58)
Female Gender [*n* (%)]	4524 (53.1)
Ethnicity [*n* (%)]	
Non-Hispanic White	3579 (42.0)
Non-Hispanic Black	1828 (21.5)
Mexican American	1624 (19.1)
Other Hispanic	770 (9.0)
Other/Mixed	717 (8.4)
Weight Status [*n* (%)]	
Normal or underweight (BMI <25.9 kg/m^2^)	3319 (39.0)
Overweight (BMI 25.9–29.9 kg/m^2^)	2480 (29.1)
Obese (BMI >30 kg/m^2^)	2719 (31.9)
PIR Status [*n* (%)]	
< 1.3	2607 (21.6)
1.3- < 3.5	2852 (34.8)
≥ 3.5	2412 (43.6)
*Missing*	*647*

**Table 2:** Geometric mean alanine aminotransferase (ALT) levels by demographic and weight status subgroups

Covariate	*N*	Mean, IU/L	95% CI for Mean		*p*-value^*^
Age					
≤ 19 years	2063	17.7	17.2	18.3	*p* < 0.0001
≥ 20 years	6455	22.2	21.9	22.6	
Gender					
Male	3994	25.1	24.6	25.7	*p* < 0.0001
Female	4524	18.9	18.6	19.2	
Race/Ethnicity					
Non-Hispanic White	3579	21.5^ab^	21.1	21.9	*p* < 0.0001
Non-Hispanic Black	1828	19.2^c^	18.8	19.7	
Mexican-American	1624	23.8^a^	23.0	24.5	
Other Hispanic	770	23.8^abc^	22.8	24.1	
Other or Mixed	717	21.5^bc^	20.6	22.5	
Weight Status [*n* (%)]					
Normal or underweight (BMI <25.9 kg/m^2^)	3319	18.5^a^	18.2	18.9	*p* < 0.0001
Overweight (BMI 25.9–29.9 kg/m^2^)	2480	22.6^b^	22.1	23.1	
Obese (BMI >30 kg/m^2^)	2719	24.6^b^	24.0	25.2	
PIR Status [*n* (%)]					
< 1.3	2607	20.4^ab^	19.9	20.9	*p* = 0.02
1.3- < 3.5	2852	21.1^a^	20.6	21.5	
≥ 3.5	2412	22.5^b^	21.9	23.0	
Missing	647	21.5^ab^	20.7	22.4	

^*^P-value from ANOVA or *t*-tests

Note: means with same superscript letter are not significantly different

**Table 3:** Geometric mean inorganic arsenic concentrations by demographic and weight status subgroups

Covariate	*N*	Mean, μg/L	95% CI for Mean		*p*-value^*^
Age					
≤ 19 years	2063	3.4	3.1	3.8	*p* = 0.03
≥ 20 years	6455	3.9	3.6	4.2	
Gender					
Male	3994	4.4	4.1	4.8	*p* < 0.0001
Female	4524	3.3	3.1	3.6	
Race/Ethnicity					
Non-Hispanic White	3579	3.3^c^	3.0	3.6	*p* < 0.0001
Non-Hispanic Black	1828	5.0^ab^	4.6	5.4	
Mexican-American	1624	4.7^a^	4.3	5.2	
Other Hispanic	770	5.7^b^	5.0	6.4	
Other or Mixed	717	5.6^ab^	4.7	6.6	
Weight Status [*n* (%)]					
Normal or underweight (BMI <25.9 kg/m^2^)	3319	3.7	3.4	4.0	*p* = 0.47
Overweight (BMI 25.9–29.9 kg/m^2^)	2480	3.9	3.5	4.3	
Obese (BMI ≥30 kg/m^2^)	2719	3.9	3.5	4.3	
PIR Status					
< 1.3	2607	3.7	3.5	4.0	*p* = 0.09
1.3- < 3.5	2852	3.6	3.3	3.9	
≥ 3.5	2412	4.0	3.6	4.4	
Missing	647	4.2	3.5	5.1	

^*^P-value from ANOVA or *t*-tests

Note: means with same superscript letter are not significantly different

In the full model, the odds of elevated ALT adjusted for age, sex, PIR, weight status, race/ethnicity and survey cycle rose with increasing arsenic exposure, from an aOR of 1.2 (95% confidence interval [CI]: 0.7,1.9) for those in the second compared to the referent (first quartile of arsenic exposure), to an aOR of 2.0 (CI: 1.2, 3.4) for those in the fourth quartile (Table 4). As testing for modification of this effect by race/ethnicity was borderline significant (F-test, *p* = 0.07), further analysis was stratified and presented for each race/ethnic group separately. aORs were highest among Mexican Americans with risk of elevated ALT, compared to the referent, rising 50% among those in the second quartile of urinary iAs concentrations [1.5 (CI: 0.95, 2.2)], 70% among those in the third quartile [1.7(CI: 1.1, 2.6)] and doubling among those in the fourth quartile [2.0 (1.3, 3.1)] (Table 4). Increased odds among non-Hispanic whites were also observed but only among those in the 4th quartile of iAs exposure. There was no indication of an increase in risk among “other Hispanics” (Table 4).

**Table 4:** Adjusted odds ratio of elevated alanine aminotransferase (ALT) levels with increasing inorganic arsenic concentrations by race/ethnicity and by weight status

Model		Quartile of Inorganic Arsenic [point estimate (95% Confidence Intervals)]							
		1st		2nd		3rd		4th	
	*N*	Cases,(%)^1^	aOR	Cases,(%)^1^	aOR (95%CI)	Cases,(%)^1^	aOR (95%CI)	Cases,(%)^1^	aOR (95%CI)
**All** ^2^	8518	506 (28%)	Ref	644 (31%)	1.2 (0.7, 1.9)	736 (33%)	1.6 (1.0, 2.8)	827 (35%)	2.0 (1.2, 3.4)
**Race/Ethnicity** ^**3**^									
Non-Hispanic White	3579	325 (30%)	Ref	310 (32%)	1.1 (0.9, 1.4)	262 (32%)	1.0 (0.8, 1.3)	261(37%)	1.4 (1.1, 1.8)
Non-Hispanic Black	1828	47 (17%)	Ref	92 (21%)	1.2 (0.8, 1.7)	127 (25%)	1.4 (0.9, 2.2)	153 (25%)	1.4 (0.9, 2.1)
Mexican Americans	1624	74 (30%)	Ref	150 (35%)	1.5 (1.0, 2.2)	202 (39%)	1.7 (1.1, 2.6)	191 (44%)	2.0 (1.3, 3.1)
Other Hispanics	770	33 (40%)	Ref	61 (39%)	0.7 (0.3, 1.3)	90 (41%)	0.9 (0.5, 1.7)	112 (36%)	0.6 (0.3, 1.1)
Other or Mixed	717	27 (24%)	Ref	31 (27%)	1.1 (0.5, 2.3)	55 (34%)	1.7 (0.7, 4.2)	110 (34%)	1.7 (0.8, 3.7)
**BMI Weight Group** ^**4**^									
Underweight/Normal	3319	146 (19%)	Ref	144 (7%)	0.9 (0.7, 1.3)	168 (20%)	1.1 (0.8, 1.5)	183 (21%)	1.34 (1.0, 1.8)
Overweight	2480	159 (31%)	Ref	183 (31%)	1.0 (0.8, 1.5)	213 (33%)	0.98 (0.7, 1.4)	262 (36%)	1.31 (0.9, 1.9)
Obese	2719	201 (39%)	Ref	317 (46%)	1.4 (1.0, 1.9)	355 (48%)	1.3 (1.0, 1.8)	382 (48%)	1.50 (1.1, 2.0)
**Obese Only** ^**4**^									
Non-Hispanic White	1067	134 (44%)	Ref	139 (47%)	1.3 (0.9, 2.1)	129 (50%)	1.3 (0.8, 2.0)	108 (50%)	1.5 (1.0, 2.2)
Non-Hispanic Black	726	17 (18%)	Ref	57 (33%)	1.7 (0.9, 3.1)	73 (37%)	2.0 (1.0, 3.9)	95 (37%)	2.0 (1.1, 3.8)
Mexican Americans	554	29 (44%)	Ref	82 (57%)	1.9 (1.0, 3.5)	102 (53%)	1.4 (0.7, 2.9)	94 (61%)	1.9 (1.0, 3.5)
Other Hispanics	255	7 (37%)	Ref	31 (57%)	2.8 (0.6, 13.4)	40 (57%)	3.3 (0.8, 13.9)	55 (49%)	1.9 (0.5, 7.5)
Other or Mixed	117	14 (50%)	Ref	8 (44%)	0.4 (0.1, 2.2)	11 (50%)	0.5 (0.1, 2.6)	30 (61%)	0.9 (0.3, 3.3)

*All* model adjusted for age, survey cycle, gender, PIR, race/ethnicity, *BMI* Weight status, and interactions (race/ethnicity*iAS, BMI weight status*iAS)

^1^%cases = # with elevated ALT divided by the total in the arsenic exposure quartile + race/ethnic or weight category subgroup

^2^Adjusted for age, survey cycle, gender, PIR, *BMI* Weight status, and race/ethnicity

^3^Adjusted for age, survey cycle, gender, PIR, and *BMI* Weight status

^4^Adjusted for age, survey cycle, gender, PIR, and race/ethnicity

Note: High blood-ALT levels were defined as >25 IU/L for boys ≤17 years and >22 IU/L for girls ≤17 years. In adults, high ALT was defined >30 IU/L in males and >19 IU/L in females

Though the interaction term for weight status by iAS was not significant (*p* = 0.4), we repeated the analysis stratified by weight category to account for the unequal distribution of obesity across the race/ethnic groups. Here the odds of elevated ALT were greater for all weight categories when iAs was highest but among those obese a threshold affect was observed with risk rising 40% at the 2nd quartile of exposure and remaining at approximately the same level through the 4th quartile. Among those obese only, increased risk of elevated ALT was observed, though not always statistically significant, in the highest iAs exposure category among all the race/ethnic groups except for the “other/mixed” race/ethnic group. The results of the sensitivity analyses using unweighted data were very similar to those obtained using data with sampling weights applied (not shown).

## Discussion

The results of our study, the first such study among humans, demonstrates an association between arsenic exposure and risk of NAFLD among U.S. adolescents and adults, an association that our results suggest varies by race/ethnicity. There was a dose-response increase in risk among Mexican Americans and an elevated risk among non-Hispanic whites with the greatest arsenic exposure, but there was no significant increase in risk among other race/ethnic groups when examining the full sample. Our stratified analysis demonstrated that risk of elevated ALT is present with high iAs exposure, regardless of an individual’s weight category, but risk occurs at lower levels of exposure among those obese. When examining this association among those obese only, we observed an increased risk at the highest quartile of iAs exposure among all race/ethnic groups except those in the “other/mixed” category.

Low chronic exposure to iAs has been associated with cardiovascular disease, [37] diabetes, [15] and sleep disturbance, [38] which are all diseases known to have oxidative stress as a key mechanism [39]. It is through increases in oxidative stress that iAs exposure may be linked to these common conditions although the exact mechanisms are unknown [40–44]. Arsenic, even at low concentrations can also activate nuclear factor (NF)-kappaB, a protein complex that controls transcription of DNA, cytokine production and cell survival [45]. Activation of NF–kappaB has been noted as a connection between oxidative stress and chronic diseases such as type 2 diabetes and insulin resistance [46, 47]. Arsenic methylation occurs in the liver, yet it is unknown how much arsenic metabolites need to accumulate in the liver to produce an ill effect [13]. It is this link that needs to be further studied to elucidate arsenic’s role in NAFLD and other chronic liver diseases.

We found a positive association between high iAs and high ALT levels, an association that was significant and strongest among Mexican Americans in the U.S. It has been shown previously that low doses of arsenic are associated with rice and poultry consumption in the U.S. [17, 23, 48, 49] Rice effectively absorbs arsenic from the soil. It is also commonly consumed by some cultures including Hispanics, African-American, and Asian cultures [49]. Although we did not assess diet, we found higher inorganic arsenic concentrations in these racial groups. Previous studies have shown arsenic levels to be higher in children that consume rice, including studies that analylzed data from the NHANES [23, 48].

This study had several important strengths. It was the first known study to examine the association between iAs exposure and NAFLD risk among humans. Use of a large, national sample with demographic and anthropometric data as well as biological samples allowed us to control for important potential confounders in our examination of this association. The availability of race/ethnicity data provided a means to assess the extent to which it modifies the association between iAs and NAFLD risk. The availability of data on high alcohol use and hepatitis B and C infection allowed us to exclude those with other known or suspected liver disease. Further, all data was collected using trained staff and standardized protocols. The laboratory methods used were of the highest quality with both arsenic and ALT measured using established in Center for Disease Control and Prevention (CDC) verified labs in the NHANES study reducing possibility of measurement error. While there were a number of samples with levels below the level of detection this had no impact on our analysis as arsenic exposure was examined in quartiles.

This study also had some limitations that must be noted. First, as this is a cross-sectional study, temporality cannot be established so the results cannot be taken to indicate causality. Secondly, a definitive diagnosis of NAFLD requires a liver biopsy, which was not available (or feasible) for this national sample. Therefore, we used ALT levels as a surrogate marker. While ALT is commonly used as a screening tool for NAFLD, there are other conditions that could contribute to a high ALT including chronic hepatitis and excessive alcohol consumption. To minimize bias due to misclassification, those with these conditions were excluded from the sample. Finally, the sample sizes in some of our subgroup analyses were very small, particularly when stratifying by obesity status, which subjects those results to a greater sampling error bias. High relative standard errors, which are reflected in wide confidence intervals, suggest that the comparison being made may not be reliable or representative. To address this, we have presented the sample size for all subgroups as well as the confidence intervals for all estimates to make the level of uncertainty around estimates clear to the reader.

## Conclusion

The results of this study demonstrate that there is a positive association between inorganic arsenic exposure and elevated ALT in humans of all weight categories, and that risk is highest among Mexican Americans and those obese, regardless of race/ethnicity. Further research is needed to confirm this association in subjects with confirmed NAFLD, to identify the biological mechanisms involved, and to develop strategies for ameliorating and reducing exposure to arsenic.

## Abbreviations

ALT: Alanine aminotransferase
Anti-HBc: Hepatitis B core antibody
Anti-HCV: Hepatitis C antibody
aOR: Adjusted odds ratio
BMI: Body mass index
iAs: Inorganic arsenic
ICP-DRC-MS: Inductively coupled-plasma dynamic reaction cell-mass spectrometry
IQR: Interquartile range
LLOD: Lower limit of detection
NAFLD: Nonalcoholic fatty liver disease
NHANES: National Health and Nutrition Examination Survey
PIR: Poverty income ratio
